# Comparing Health Care Utilization Between Medicare Advantage and Traditional Medicare by Dementia Status: A Propensity‐Weighted Analysis

**DOI:** 10.1111/1475-6773.70174

**Published:** 2026-10-15

**Authors:** Jason M. Joseph, Jennifer Rachel, Gang Han, Matthew Lee Smith

**Affiliations:** ^1^ Department of Epidemiology and Biostatistics Texas A&M School of Public Health College Station Texas USA; ^2^ Department of Community Medicine Aarupadai Veedu Medical College Kirumampakkam Puducherry India; ^3^ Department of Applied Health Science School of Public Health, Indiana University – Bloomington Bloomington Indiana USA

**Keywords:** Alzheimer disease and related dementias, health care utilization, Medicare advantage, Medicare current beneficiary survey, propensity score weighting, traditional Medicare

## Abstract

**Objective:**

To compare health care utilization between Medicare Advantage (MA) and Traditional Medicare (TM) beneficiaries, stratified by Alzheimer's disease and related dementias (ADRD) status.

**Study Setting and Design:**

Repeated cross‐sectional observational analysis of community‐dwelling Medicare beneficiaries aged ≥ 65 years continuously enrolled in MA or TM. Eight self‐ or proxy‐reported binary utilization outcomes over a 12‐month reference period were estimated by survey‐weighted linear probability models stratified by ADRD status.

**Data Sources and Analytic Sample:**

Secondary analysis of pooled 2018–2022 Medicare Current Beneficiary Survey (MCBS) public use files (*N* = 43,995: 974 TM and 954 MA with ADRD; 23,050 TM and 19,017 MA without ADRD), excluding beneficiaries entitled through disability or end‐stage renal disease and records with missing covariates. Dual‐eligible beneficiaries were retained and dual eligibility included as a covariate. Stabilized inverse probability of treatment weights multiplied by MCBS full‐sample weights addressed non‐random MA enrollment.

**Principal Findings:**

Among beneficiaries with ADRD, seven of eight adjusted MA − TM differences were not statistically significant. Specialist visits were more common among MA beneficiaries with ADRD (5.2 percentage points; 95% CI, −0.1 to 10.2). Among beneficiaries without ADRD, MA was associated with lower outpatient hospital visits (−2.6; −3.7 to −1.6) and lower reported need for home health visits (−0.9; −1.5 to −0.3). Effect modification by ADRD status was significant only for specialist visits (interaction 6.3 percentage points; *p* = 0.004).

**Conclusions:**

Adjusted health care utilization was broadly comparable between MA and TM for beneficiaries with ADRD, with the exception of higher specialist use in MA. Among beneficiaries without ADRD, MA was associated with lower outpatient hospital use. Plan‐type differences were smaller and fewer for beneficiaries with ADRD.

## Keypoints

- What is known on this topic?
  ○ Over 7 million older adults in the United States live with dementia, with Medicare and Medicaid financing the majority of associated care expenditures.
  ○ More than half of Medicare beneficiaries are now enrolled in Medicare Advantage rather than Traditional Medicare, but evidence comparing utilization for people with dementia is limited.
  ○ Prior comparisons relied on older data or did not account for the fact that beneficiaries do not enroll in Medicare Advantage at random, which can bias plan‐type differences
- What this study found?
  ○ After accounting for plan selection, adjusted health care utilization was broadly similar between Medicare Advantage and Traditional Medicare for community‐dwelling beneficiaries with dementia.
  ○ The exception was specialist care: beneficiaries with dementia enrolled in Medicare Advantage had higher specialist visit use than those in Traditional Medicare (interaction *p* = 0.004). Among beneficiaries without dementia, Medicare Advantage was associated with lower outpatient hospital use and modestly lower reported need for home health services.

## 1 Introduction

Alzheimer's disease and related dementias (ADRD) pose a growing public health challenge in the United States. Over 7 million older adults currently live with ADRD, and this number is projected to nearly double by 2060 in the absence of effective preventive or curative interventions [1]. ADRD is frequently underdiagnosed or identified late, limiting timely management and support [2]. Caring for individuals with ADRD generates substantial economic burden: Medicare and Medicaid finance roughly two‐thirds of total ADRD‐related expenditures, which exceed $360 billion annually [1, 3]. Medicare, the largest insurer of older adults in the United States, offers two primary coverage options: Traditional fee‐for‐service Medicare (TM) and Medicare Advantage (MA), the latter of which provides benefits through privately administered managed care plans [4].

Under MA's capitated, risk‐adjusted payment model, plans are incentivized to coordinate care and contain costs [5]. As of 2025, more than half of eligible Medicare beneficiaries (54%) were enrolled in MA plans, although the rate of MA enrollment growth has recently slowed [6]. The remaining beneficiaries enrolled in TM continue to access services on a fee‐for‐service basis, with broad provider choice but without the capitated care coordination structures that characterize MA. National data show that TM beneficiaries have historically reported higher per‐capita inpatient and outpatient utilization than MA enrollees [7], reflecting differences in benefit design, network breadth, and beneficiary selection. Comparative studies have similarly reported that MA beneficiaries experience lower hospital readmission rates [8], better patient experiences [9], and lower overall health care utilization than TM beneficiaries [10].

Policy changes over the past decade, along with adaptations by MA plans, have contributed to improved utilization patterns in MA compared with TM [11]. Given the increasing number of beneficiaries with ADRD enrolling in MA, these plans may have unique incentives and opportunities to strengthen care coordination and disease management for this population [12]. Prior work has shown that following a new ADRD diagnosis, TM beneficiaries were more likely to transition into MA, while those already enrolled in MA were more likely to remain in their plans [13]. This pattern suggests that MA may be viewed as a favorable coverage option for individuals with ADRD and their caregivers, although empirical evidence on outcomes specific to this population remains limited [14, 15].

While previous studies have compared ADRD‐related care between MA and TM using administrative claims and survey data, important gaps remain [8]. Jacobson and colleagues used a difference‐in‐differences design to examine short‐term utilization changes surrounding a new dementia diagnosis [14]. Park and colleagues used MCBS survey waves from 2010 to 2016 to compare utilization and health status between MA and TM beneficiaries with and without ADRD but excluded dual‐eligible beneficiaries, did not formally test for effect modification by ADRD status, and did not report structured sensitivity analyses [15]. To build on this evidence, the present study uses pooled 2018–2022 Medicare Current Beneficiary Survey (MCBS) data—a contemporary window during which MA enrollment surpassed 50% of eligible beneficiaries and coincided with COVID‐19‐era disruptions in care patterns—to examine differences in health care utilization between beneficiaries with and without ADRD across MA and TM enrollment.

## 2 Methods

We conducted a repeated cross‐sectional analysis using deidentified public use files from the Medicare Current Beneficiary Survey (MCBS), pooling survey years 2018 through 2022. The MCBS draws a nationally representative sample of Medicare enrollees through a stratified, multistage area‐probability design [16]. Sampled beneficiaries complete structured, in‐person interviews at enrollment and annual follow‐up rounds, generating individual‐level data on demographics, health status, chronic conditions, functional limitations, utilization, and payment sources. The MCBS links survey responses with Medicare administrative records, providing validated utilization measures against fee‐for‐service claims for TM enrollees [17]. We pooled five survey years to increase statistical power for subgroup analyses, particularly among beneficiaries with ADRD. For this study, Institutional Review Board approval was not sought given the use of secondary, publicly available data.

### 2.1 Study Population

The analytic sample comprised Medicare beneficiaries aged 65 years or older with at least 12 months of continuous enrollment in Medicare Parts A and B in either MA or TM. Beneficiaries with only partial‐year Medicare coverage, including those who aged into Medicare mid‐year at Age 65, were excluded. We further excluded individuals whose original Medicare entitlement was based on disability or end‐stage renal disease, as these groups enter the program through different pathways and exhibit distinct patterns of plan selection and service use [18]. Dually eligible beneficiaries were retained in the analytic sample and dual eligibility was included as a covariate in both the propensity score model and outcome regressions. After exclusions and a complete‐case restriction on propensity model covariates, the final analytic sample comprised 43,995 beneficiaries (eFigure 1).

### 2.2 Measures

The primary exposure was 12‐month enrollment in MA (Coded 1) versus TM (Coded 0). ADRD status was ascertained from the survey item, “Has a doctor ever told you that you had Alzheimer's disease or dementia?” Beneficiaries responding affirmatively were classified as having ADRD. Based on these items, four mutually exclusive subgroups were created: (a) MA beneficiaries with ADRD; (b) TM beneficiaries with ADRD; (c) MA beneficiaries without ADRD; and (d) TM beneficiaries without ADRD.

The primary outcomes were self‐ or proxy‐reported measures of health care utilization across eight service categories: medical practitioner visits; emergency room visits; outpatient hospital visits; home health visits; prescription drug fills (a single fill of a single medication in a single container); specialist visits; hospital admissions; and dental visits. Each outcome was modeled as a binary indicator (any vs. none) over the 12‐month survey reference period. Unadjusted survey‐weighted prevalences are reported in eTable 4. For TM beneficiaries, self‐reported utilization has been validated against Medicare claims [17].

Covariates included age (65–74, 75+ years), sex (male, female), race and ethnicity (non‐Hispanic White, non‐Hispanic Black, Hispanic, other), educational attainment (less than high school, high school/GED, more than high school), marital status (married, widowed, divorced/separated, never married), income relative to the federal poverty level (< 100%, 100%–199%, 200%–299%, 300%–399%, ≥ 400%), metropolitan residence (yes, no), survey year (2018–2022), total activities of daily living (ADL) limitations (no ADL, IADLs only, 1–2, 3–4, and 5–6 ADLs), and dual eligibility (non‐dual, part‐year dual, full‐year dual). Chronic conditions were ascertained from self‐reported physician diagnoses (“Has a doctor ever told you that you had …”) and included heart failure, diabetes, hypertension, stroke, emphysema/asthma/COPD, hardening of arteries, mental disorder, osteoporosis, cancer, and complete/partial paralysis (each yes/no). Records with missing covariate data were excluded; the final analytic sample reflects complete cases.

### 2.3 Statistical Analysis

We first examined unadjusted characteristics and utilization patterns across MA and TM enrollees, stratified by ADRD status, using Rao‐Scott survey‐adjusted chi‐square tests for categorical variables. MCBS survey weights were applied throughout to produce nationally representative estimates.

Because enrollment in MA is not random, naïve comparisons would be biased by self‐selection on observable and unobservable characteristics [19]. We therefore applied inverse probability of treatment weighting (IPTW) to balance measured covariates across plan groups while preserving the full analytic sample [20]. Propensity scores for MA enrollment were estimated via logistic regression including all listed covariates. A pooled model including ADRD status was used due to separation issues in stratum‐specific models. Stabilized weights were computed as the marginal probability of the observed treatment divided by the estimated conditional probability of that treatment given covariates: for MA enrollees, sw_i_ = Pr(MA)/Pr(MA|*X*_i_); for TM enrollees, sw_*i*_ = [1 − Pr(MA)]/[1 − Pr(MA|*X*_*i*_)], where Pr(MA|*X*_*i*_) is the estimated propensity score [21]. Stabilized IPTW weights were multiplied by MCBS survey weights to produce final analytic weights [22].

Propensity score overlap was examined graphically (eFigure 2), and the distribution of analytic weights is summarized in eTable 3. Covariate balance was assessed using standardized mean differences (SMDs), with values below 0.10 indicating adequate balance (eTable 1; eFigure 3) [23]. Utilization outcomes were estimated using survey‐weighted linear probability models stratified by ADRD status, with MA enrollment as the primary independent variable. We used survey‐weighted linear probability models as the primary specification because linear probability model coefficients are directly interpretable as absolute risk differences, which is the estimand of policy interest for this comparison; the LPM avoids the noncollapsibility complications that arise in interpreting IPTW‐adjusted odds ratios; and prior MCBS‐based utilization studies of MA versus TM have used comparable specifications.

Both unadjusted and IPTW‐adjusted results are reported as weighted proportions and mean differences with 95% confidence intervals are reported in Table 2. Confidence intervals for the MA − TM differences were constructed by nonparametric bootstrap with 1000 replicates, preserving the estimated IPTW weights within each resample. Variance for interaction tests and remaining survey‐weighted regression estimates was estimated using balanced repeated replication with the MCBS 100 replicate weights and Fay's adjustment (*ρ* = 0.3), following the CMS‐recommended variance estimation procedure for the MCBS complex sampling design. To formally test whether MA–TM utilization differences varied by ADRD status, we estimated pooled models including an effect modification by ADRD status (Table 3).

As a sensitivity analysis, we re‐estimated each utilization model using survey‐weighted logistic regression, reporting odds ratios and average marginal effects (eTable 6), and calculated E‐values for each statistically significant IPTW‐adjusted association to quantify the strength of unmeasured confounding required to fully explain the observed effect (eTable 2) [24]. All analyses were performed in SAS Enterprise Guide version 8.3 (SAS Institute, Cary, NC) and R version 4.3. Hypothesis tests were two‐sided, with *p* < 0.05 considered statistically significant.

## 3 Results

The final analytic sample comprised 43,995 Medicare beneficiaries aged 65 years and older: 23,050 TM and 19,017 MA beneficiaries without ADRD, and 974 TM and 954 MA beneficiaries with ADRD (Table 1; eFigure 1). Among beneficiaries with ADRD, MA and TM enrollees had similar age distributions, with most aged 75 years or older (84% TM, 85% MA), although MA enrollees included a higher proportion of women (63% vs. 57%; SMD = 0.113). Substantial differences were observed in race and ethnicity (SMD = 0.364), with greater representation of non‐Hispanic Black (12% vs. 7.3%) and Hispanic (23% vs. 12%) beneficiaries in MA. MA enrollees with ADRD were also more likely to live in metropolitan areas (86% vs. 74%; SMD = 0.296), have incomes below 100% of the federal poverty level (25% vs. 22%; SMD = 0.280), and report less than a high school education (33% vs. 27%; SMD = 0.173). Comorbidity profiles differed modestly across plan types. A similar pattern of socioeconomic and demographic imbalance was observed among beneficiaries without ADRD (race and ethnicity SMD = 0.351, income SMD = 0.353, education SMD = 0.275, residence SMD = 0.258), with comorbidity profiles closely aligned across plan types (all SMDs ≤ 0.10). Despite these differences and the greater socioeconomic vulnerability among MA beneficiaries, MA enrollees without ADRD were not uniformly healthier than their TM counterparts. Proxy respondents accounted for 63.7% of ADRD interviews and 6.5% of non‐ADRD interviews.

**TABLE 1:** Sample characteristics of traditional Medicare and Medicare advantage beneficiaries with and without ADRD.

Characteristic	With ADRD			Without ADRD		
	TM (*n* = 974)	MA (*n* = 954)	SMD	TM (*n* = 23,050)	MA (*n* = 19,017)	SMD
Age, years			0.028			0.046
65–74	156 (16.0%)	143 (15.0%)		9868 (42.8%)	7712 (40.6%)	
75+	818 (84.0%)	811 (85.0%)		13,182 (57.2%)	11,305 (59.4%)	
Sex			0.113			0.064
Male	415 (42.6%)	354 (37.1%)		10,594 (46.0%)	8132 (42.8%)	
Female	559 (57.4%)	600 (62.9%)		12,456 (54.0%)	10,885 (57.2%)	
Race and ethnicity			0.364			0.351
Non‐Hispanic White	720 (73.9%)	566 (59.3%)		19,225 (83.4%)	13,347 (70.2%)	
Non‐Hispanic Black	71 (7.3%)	116 (12.2%)		1249 (5.4%)	2008 (10.6%)	
Hispanic	118 (12.1%)	221 (23.2%)		1390 (6.0%)	2665 (14.0%)	
Other	65 (6.7%)	51 (5.3%)		1186 (5.1%)	997 (5.2%)	
Education			0.173			0.275
Less than high school	262 (26.9%)	319 (33.4%)		2266 (9.8%)	3387 (17.8%)	
High school/GED	315 (32.3%)	318 (33.3%)		6484 (28.1%)	6034 (31.7%)	
More than high school	397 (40.8%)	317 (33.2%)		14,300 (62.0%)	9596 (50.5%)	
Income relative to FPL			0.280			0.353
< 100% FPL	209 (21.5%)	239 (25.1%)		1910 (8.3%)	2814 (14.8%)	
100–199%	52 (5.3%)	91 (9.5%)		811 (3.5%)	1147 (6.0%)	
200–299%	45 (4.6%)	43 (4.5%)		593 (2.6%)	838 (4.4%)	
300–399%	158 (16.2%)	202 (21.2%)		3020 (13.1%)	3547 (18.7%)	
≥ 400%	510 (52.4%)	379 (39.7%)		16,716 (72.5%)	10,671 (56.1%)	
Marital status			0.198			0.133
Married	459 (47.1%)	404 (42.3%)		12,622 (54.8%)	9384 (49.3%)	
Widowed	396 (40.7%)	367 (38.5%)		6316 (27.4%)	5330 (28.0%)	
Divorced/separated	98 (10.1%)	143 (15.0%)		2993 (13.0%)	3238 (17.0%)	
Never married	21 (2.2%)	40 (4.2%)		1119 (4.9%)	1065 (5.6%)	
Residence			0.296			0.258
Metropolitan	722 (74.1%)	819 (85.8%)		16,964 (73.6%)	15,985 (84.1%)	
Non‐metropolitan	252 (25.9%)	135 (14.2%)		6086 (26.4%)	3032 (15.9%)	
Total ADL limitations			0.077			0.029
No ADL	182 (18.7%)	151 (15.8%)		14,869 (64.5%)	12,044 (63.3%)	
IADLs only	188 (19.3%)	194 (20.3%)		2868 (12.4%)	2383 (12.5%)	
1–2 ADLs	241 (24.7%)	245 (25.7%)		3862 (16.8%)	3361 (17.7%)	
3–4 ADLs	163 (16.7%)	167 (17.5%)		1023 (4.4%)	886 (4.7%)	
5–6 ADLs	200 (20.5%)	197 (20.6%)		428 (1.9%)	343 (1.8%)	
Dual eligibility			0.225			0.286
Non‐dual	759 (77.9%)	650 (68.1%)		21,293 (92.4%)	15,800 (83.1%)	
Part‐year dual	15 (1.5%)	27 (2.8%)		123 (0.5%)	230 (1.2%)	
Full‐year dual	200 (20.5%)	277 (29.0%)		1634 (7.1%)	2987 (15.7%)	
Comorbidity (yes)						
Heart failure	120 (12.3%)	131 (13.7%)	0.042	1440 (6.2%)	1158 (6.1%)	0.007
Diabetes	320 (32.9%)	350 (36.7%)	0.081	6883 (29.9%)	6498 (34.2%)	0.092
Hypertension	689 (70.7%)	695 (72.9%)	0.047	14,961 (64.9%)	12,965 (68.2%)	0.069
Stroke	247 (25.4%)	258 (27.0%)	0.038	2134 (9.3%)	1798 (9.5%)	0.007
Emphysema/asthma/COPD	198 (20.3%)	237 (24.8%)	0.108	4093 (17.8%)	3416 (18.0%)	0.005
Hardening of arteries	135 (13.9%)	141 (14.8%)	0.026	2002 (8.7%)	1447 (7.6%)	0.039
Mental disorder	122 (12.5%)	104 (10.9%)	0.051	839 (3.6%)	703 (3.7%)	0.003
Osteoporosis	282 (29.0%)	272 (28.5%)	0.010	4444 (19.3%)	3681 (19.4%)	0.002
Cancer	205 (21.0%)	211 (22.1%)	0.026	4998 (21.7%)	3866 (20.3%)	0.033
Complete/partial paralysis	51 (5.2%)	44 (4.6%)	0.029	500 (2.2%)	460 (2.4%)	0.017
Survey year			0.168			0.137
2018	254 (26.1%)	206 (21.6%)		4874 (21.1%)	3371 (17.7%)	
2019	234 (24.0%)	202 (21.2%)		4765 (20.7%)	3629 (19.1%)	
2020	199 (20.4%)	197 (20.6%)		5083 (22.1%)	3986 (21.0%)	
2021	145 (14.9%)	169 (17.7%)		4312 (18.7%)	3925 (20.6%)	
2022	142 (14.6%)	180 (18.9%)		4016 (17.4%)	4106 (21.6%)	

*Note:* Values are *n* (%). SMD denotes the standardized mean difference between MA and TM beneficiaries computed within each ADRD stratum; SMDs ≤ 0.10 indicate adequate balance. For categorical variables with more than two levels, SMD is the multinomial generalization [25]. Comorbidities reflect self‐reported physician diagnosis (“Has a doctor ever told you that you had …”).

Abbreviations: ADL, activities of daily living; ADRD, Alzheimer disease and related dementias; FPL, federal poverty level; GED, General Educational Development; MA, Medicare Advantage; SMD, standardized mean difference; TM, Traditional Medicare.

Unadjusted survey‐weighted prevalences of any use (eTable 4) revealed differences between TM and MA enrollees across most service categories, with directional patterns broadly consistent with the IPTW‐adjusted estimates reported below. After applying stabilized IPTW combined with MCBS survey weights, all covariate SMDs were reduced below 0.05 (eTable 1; eFigure 3), supporting the validity of weighted comparisons. The distribution of analytic weights is summarized in eTable 3, and propensity score overlap between plan groups is shown in eFigure 2.

Table 2 presents IPTW‐adjusted prevalences of any use and MA − TM differences, stratified by ADRD status. Among beneficiaries with ADRD, adjusted utilization rates were largely comparable across plan types, with no statistically significant differences for medical practitioner visits (TM 92.7% vs. MA 91.5%; difference −1.2 percentage points [95% CI, −4.1 to 1.6]), emergency department visits (37.1% vs. 37.2%; 0.1 [−5.2 to 5.4]), outpatient hospital visits (29.8% vs. 28.1%; −1.7 [−6.6 to 3.4]), home health visits (35.8% vs. 32.0%; −3.8 [−9.2 to 1.7]), prescription drug fills (98.7% vs. 98.4%; −0.2 [−1.5 to 1.0]), hospital admissions (25.1% vs. 22.0%; −3.1 [−8.7 to 2.7]), or dental visits (11.1% vs. 10.3%; −0.9 [−3.9 to 2.4]). One difference reached borderline significance: specialist visits were more common among MA beneficiaries with ADRD (66.4% vs. 61.2%; 5.2 [−0.1 to 10.2]), a 5.2‐percentage‐point absolute increase corresponding to approximately 8.5% higher specialist visit prevalence relative to the TM baseline of 61.2%.

**TABLE 2:** IPTW‐adjusted prevalence and MA − TM differences in health care utilization outcomes among traditional Medicare and Medicare advantage beneficiaries with and without ADRD.

Outcome	TM %	MA %	MA − TM difference, % (95% CI)	TM %	MA %	MA − TM difference, % (95% CI)
Medical practitioner visit	92.7	91.5	−1.2 (−4.1 to 1.6)	91.6	91.3	−0.2 (−0.9 to 0.4)
Emergency department visit	37.1	37.2	0.1 (−5.2 to 5.4)	20.8	20.8	0.0 (−0.9 to 0.9)
Outpatient hospital visit	29.8	28.1	−1.7 (−6.6 to 3.4)	31.1	28.6	−2.6 (−3.7 to −1.6)
Home health visits	35.8	32.0	−3.8 (−9.2 to 1.7)	7.5	6.6	−0.9 (−1.5 to −0.3)
Prescription drug fill	98.7	98.4	−0.2 (−1.5 to 1.0)	95.6	95.7	0.2 (−0.4 to 0.6)
Specialist visit	61.2	66.4	5.2 (−0.1 to 10.2)	68.2	67.1	−1.1 (−2.1 to 0.1)
Hospital admission	25.1	22.0	−3.1 (−8.7 to 2.7)	15.4	15.0	−0.4 (−1.4 to 0.4)
Dental care visits	11.1	10.3	−0.9 (−3.9 to 2.4)	8.5	8.9	0.4 (−0.3 to 1.0)

*Note:* Values are IPTW‐adjusted prevalences of any use versus none (%) and paired MA − TM differences (percentage points). Estimates are from survey‐weighted linear probability models applying stabilized inverse probability of treatment weights multiplied by Medicare Current Beneficiary Survey full‐sample weights. Ninety‐five percent confidence intervals for MA − TM differences were obtained by nonparametric bootstrap with 1000 replicates, preserving the estimated IPTW weights within each resample. Models adjusted for age, sex, race and ethnicity, education, income, marital status, residence, total ADL limitations, dual eligibility, survey year, and the comorbidities listed in Table 1. Unadjusted survey‐weighted prevalences are reported in eTable 4.

Abbreviations: ADL, activities of daily living; ADRD, Alzheimer disease and related dementias; CI, confidence interval; IPTW, inverse probability of treatment weighting; MA, Medicare Advantage; TM, Traditional Medicare.

Among beneficiaries without ADRD, plan‐type differences were smaller in magnitude but more precisely estimated. Outpatient hospital visits were significantly less common in MA (28.6% vs. 31.1%; difference −2.6 [95% CI, −3.7 to −1.6]); home health visits were modestly lower in MA (6.6% vs. 7.5%; −0.9 [−1.5 to −0.3]). Differences for medical practitioner, emergency department, specialist, prescription drug fill, hospital admission, and dental visits outcomes were small and not statistically significant. Effect modification by ADRD status (Table 3) was statistically significant only for specialist visits (interaction 6.3 percentage points; *p* = 0.004), confirming that MA enrollment was associated with higher specialist use among beneficiaries with ADRD but not among those without ADRD. Overall, the adjusted results indicate that plan‐type differences in utilization were fewer in number and smaller in magnitude among beneficiaries with ADRD; the more pronounced MA − TM differences observed in the non‐ADRD population are consistent with the pattern suggested by the unadjusted comparisons.

**TABLE 3:** MA × ADRD interaction estimates from pooled survey‐weighted linear probability models.

Outcome	MA − TM difference (without ADRD), %	MA − TM difference (with ADRD), %	Interaction (%, 95% CI)	*p*
Medical practitioner visit	−0.2	−1.2	−1.0 (−3.3 to 1.4)	0.42
Emergency department visit	0.0	0.1	0.1 (−3.9 to 4.1)	0.96
Outpatient hospital visit	−2.6	−1.7	0.9 (−3.0 to 4.9)	0.65
Home health visits	−0.9	−3.8	−2.9 (−7.5 to 1.7)	0.21
Prescription drug fill	0.2	−0.2	−0.4 (−1.5 to 0.7)	0.49
Specialist visit	−1.1	5.2	6.3 (2.1 to 10.5)	0.004
Hospital admission	−0.4	−3.1	−2.7 (−6.8 to 1.4)	0.20
Dental care visits	0.4	−0.9	−1.3 (−4.0 to 1.5)	0.37

*Note:* Values are MA × ADRD interaction estimates (percentage points) from pooled survey‐weighted linear probability models fit to the full analytic sample, adjusted for all Table 1 covariates and applying stabilized IPTW weights. *p*‐values are from balanced repeated replication using the Medicare Current Beneficiary Survey 100 replicate weights and Fay's adjustment (*ρ* = 0.3).

Abbreviations: ADRD, Alzheimer disease and related dementias; CI, confidence interval; IPTW, inverse probability of treatment weighting; MA, Medicare Advantage; TM, Traditional Medicare.

Sensitivity analyses supported the primary findings. E‐values for the statistically significant IPTW‐adjusted associations (eTable 2) ranged from approximately 1.4 to 2.3 for point estimates and from approximately 1.2 to 1.6 for the lower confidence bounds. This indicates that an unmeasured confounder would need to be associated with both MA enrollment and the outcome by risk ratios of these magnitudes, above and beyond the covariates already balanced by IPTW, to fully explain the observed associations. Logistic regression sensitivity (eTable 6) yielded odds ratios and average marginal effects directionally consistent with the LPM estimates for all eight outcomes. A duals‐stratified sensitivity (eTable 5) re‐estimating MA − TM differences separately among non‐dual and any‐dual beneficiaries within each ADRD stratum was directionally consistent with the pooled analysis.

## 4 Discussion

In this nationally representative analysis of Medicare beneficiaries, drawn from five consecutive waves of the MCBS, we examined differences in health care utilization between beneficiaries with and without ADRD across MA and TM enrollment types. We found that plan‐type differences in utilization were substantially attenuated among beneficiaries with ADRD, with one notable exception: adjusted specialist visits were more common among MA enrollees with ADRD than among their TM counterparts, and the MA‐versus‐TM difference in specialist use was significantly more positive in the ADRD stratum than in the non‐ADRD stratum (interaction *p* = 0.004). Among beneficiaries without ADRD, MA enrollment was associated with significantly lower outpatient hospital utilization and a small but statistically detectable reduction in home health visits, while most other service categories did not differ materially between plan types after adjustment.

Consistent with existing evidence about MA enrollment patterns, we found that MA and TM beneficiaries with ADRD exhibited broadly similar demographic and clinical profiles, suggesting limited differences in case mix across plan types for this high‐need population [26]. Beneficiaries without ADRD showed greater socioeconomic differences between MA and TM, with MA enrollees more likely to be from racially and ethnically diverse and lower‐income backgrounds; patterns consistent with national trends in MA growth, which has been disproportionately concentrated among historically underserved populations [15, 27]. These differences do not necessarily reflect favorable risk selection into MA, consistent with recent evidence finding limited health‐based selection in MA enrollment [28]. The similarity in clinical profiles among ADRD beneficiaries is significant, particularly in light of previous research indicating a surge in MA enrollment post‐diagnosis. This trend likely reflects the belief among patients and caregivers that MA plans provide adaptable, supplemental benefits capable of addressing complex medical, behavioral, and social needs [29, 30].

Our findings align with a growing body of literature demonstrating that MA enrollees generally experience fewer inpatient and outpatient encounters and have lower overall utilization compared with TM beneficiaries [31]. Prior studies have similarly reported reduced access to certain outpatient services in MA, reflecting differences in provider network structure, prior authorization requirements, and plan‐level care management strategies [32]. Evidence specific to ADRD populations has been more limited, but existing work suggests that individuals with higher clinical complexity or functional impairment often exhibit more comparable care patterns across MA and TM, a trend consistent with our results for the majority of the outcomes we examined [8, 33]. Our observation of higher specialist visit rates among MA enrollees with ADRD extends this literature by suggesting that MA care‐management infrastructure—including chronic‐condition Special Needs Plans and dementia‐specific supplemental benefits—may facilitate specialist engagement for beneficiaries with cognitive impairment, in contrast to the more restrictive specialty access patterns often observed in the general MA population.

Several mechanisms may explain the observed plan‐type differences in utilization. MA plans commonly employ narrower provider networks, step‐therapy requirements, and utilization management tools that may curtail the use of outpatient specialty and hospital services, particularly among beneficiaries without high acute care needs [27, 34, 35]. These structural features may account for the significantly lower outpatient hospital utilization observed among MA enrollees without ADRD in this study [5, 27]. Conversely, beneficiaries with ADRD who typically require frequent clinical contact, longitudinal care coordination, and multidisciplinary management may receive more consistent services regardless of plan type, potentially explaining the attenuated plan‐type differences observed for most outcomes in this subgroup [3, 36]. The greater specialist use observed among MA enrollees with ADRD likely reflects MA plans' emphasis on care coordination, referral pathways, and supplemental benefits targeted at complex chronic‐condition populations, which may facilitate specialist access for beneficiaries with cognitive impairment despite the network and prior‐authorization constraints that typically restrict specialty care in the broader MA population [37, 38].

These findings have several implications for Medicare policy and program oversight. The broadly comparable adjusted utilization observed across MA and TM among beneficiaries with ADRD suggests that plan structure may exert a limited differential effect on routine service use for this clinically complex group; however, the higher adjusted specialist use among MA enrollees with ADRD warrants further examination to determine whether the additional specialist contact reflects better care coordination, marketing‐driven upcoding of specialist visits, or unmet dementia‐specific specialty need not captured in TM claims [39]. Among beneficiaries without ADRD, the consistent pattern of lower outpatient hospital use in MA is consistent with MA's emphasis on care management and benefit design but may also reflect a combination of efficient substitution and access constraints [40].

CMS should require MA plans to report utilization of specialty and other ancillary services stratified by chronic conditions including ADRD, either as a Star Ratings measure or through a comparable disclosure framework, to surface gaps between marketed and realized benefit access [3, 41]. MedPAC should evaluate whether current MA network adequacy standards adequately reflect the specialty and geographic care needs of beneficiaries with ADRD, given that MA growth has been concentrated in metropolitan areas while ADRD prevalence is distributed nationally. Prior authorization and step‐therapy requirements warrant review for appropriate calibration to clinically complex subgroups; the comparable utilization we observed for most outcomes among beneficiaries with ADRD suggests that current management practices have not generated large differential access barriers in this group, but the consistently lower outpatient hospital use among MA enrollees without ADRD raises questions about whether utilization management may be more restrictive than clinically warranted in lower‐acuity populations [11, 19].

For research, the most informative next step is a linkage of MCBS responses to Medicare administrative encounter and Part D claims data to assess longitudinal care trajectories before and after dementia diagnosis, with attention to whether observed plan‐type differences widen or attenuate as ADRD severity progresses and whether utilization differs across MA Special Needs Plans (SNPs) designed for institutional or chronic‐condition populations.

## 5 Limitations

Our study has several limitations. First, all utilization outcomes were derived from self‐ or proxy‐reported MCBS data, which may be subject to recall bias and misclassification, particularly for cognitively impaired beneficiaries whose responses are commonly provided by proxies; a higher proxy rate in the ADRD subgroup (63.7% vs. 6.5% in the non‐ADRD subgroup) may result in differential measurement error across strata. Second, encounter data submission from MA plans to CMS has historically been incomplete, which may lead to systematic underreporting of MA service use; for this reason, our primary inference focuses on the IPTW‐adjusted any‐vs‐none rates (Table 2), which are less sensitive to undercount of repeat encounters than continuous visit‐count outcomes. Third, ADRD ascertainment relied on a single self‐ or proxy‐reported survey item and does not permit differentiation by dementia subtype or severity. The resulting sample prevalence (approximately 4.4%) is lower than claims‐based estimates in the Medicare population aged 65 and older (approximately 11%–12%), reflecting well‐documented survey under‐report of dementia. Assuming this misclassification is non‐differential with respect to plan type, it would attenuate our MA × ADRD interaction estimates toward the null. Survey weights adjust for the complex sampling design and unit non‐response but not for undiagnosed ADRD in the underlying population. Fourth, despite IPTW, residual confounding from unmeasured factors including cognitive impairment severity, informal caregiver involvement, plan‐specific benefit designs, and local health system characteristics cannot be excluded; *E*‐values (eTable 2) suggest that moderate unmeasured confounding would be required to fully explain each observed adjusted association. Fifth, the repeated cross‐sectional design precludes causal inference and does not permit assessment of temporal changes in care patterns. Sixth, plan‐level heterogeneity within MA—including differences in benefit design, provider network breadth, prior‐authorization requirements, and utilization management—cannot be characterized in the MCBS Public Use Files, which do not identify specific MA plan types (such as HMO vs. PPO or Special Needs Plans) or release beneficiary state and territory of residence; our estimates therefore reflect an average across MA plan configurations and cannot separately identify beneficiaries residing in Puerto Rico.

## Funding

The authors have nothing to report.

## Conflicts of Interest

The authors declare no conflicts of interest.

## Supporting information


**eTable 1.** Covariate balance before and after inverse probability of treatment weighting.
**eTable 2.** E‐value sensitivity analysis for unmeasured confounding.
**eTable 3.** Distribution of analytic weights.
**eTable 4.** Duals‐stratified sensitivity for MA‐TM utilization differences.
**eTable 5.** Logistic regression sensitivity (odds ratios and average marginal effects).
**eTable 6.** Variable source and definition table.
**eFigure 1.** Study flow diagram (CONSORT).
**eFigure 2.** Propensity score distributions by Medicare plan type.
**eFigure 3.** Love plot of covariate balance before and after IPTW.

## Data Availability

The data that supports the findings of this study are openly available from the Centers for Medicare & Medicaid Services through the Medicare Current Beneficiary Survey Public Use File at https://www.cms.gov/data‐research/research/medicare‐current‐beneficiary‐survey‐mcbs.
