# Supplementary material for: Comparing Health Care Utilization Between Medicare Advantage and Traditional Medicare by Dementia Status: A Propensity‐Weighted Analysis
Source: Health Serv Res. 2026 Oct 15;61(5):e70174. doi: 10.1111/1475-6773.70174 (PMC13673132; doi:10.1111/1475-6773.70174)
Supplement: Supplementary file 1 [file HESR-61-e70174-s001.docx]

**Supplementary Materials**

*Comparing Health Care Utilization Between Medicare Advantage and Traditional Medicare by Dementia Status: A Propensity-Weighted Analysis*

**Contents**

| **Item** | **Description** |
| --- | --- |
| **eTable 1** | Covariate Balance Before and After Inverse Probability of Treatment Weighting |
| **eTable 2** | E-Value Sensitivity Analysis for Unmeasured Confounding |
| **eTable 3** | Distribution of Analytic Weights |
| **eTable 4** | Duals-Stratified Sensitivity for MA-TM Utilization Differences |
| **eTable 5** | Logistic Regression Sensitivity (Odds Ratios and Average Marginal Effects) |
| **eTable 6** | Variable Source and Definition Table |
| **eFigure 1** | Study Flow Diagram (CONSORT) |
| **eFigure 2** | Propensity Score Distributions by Medicare Plan Type |
| **eFigure 3** | Love Plot of Covariate Balance Before and After IPTW |

**eTable 1. Covariate Balance Before and After Inverse Probability of Treatment Weighting**

***Panel A: Overall Sample***

| **Covariate** | **Unweighted SMD** | **IPTW-Weighted SMD** | **Balance** |
| --- | --- | --- | --- |
| Age | 0.049 | 0.001 | Excellent |
| Sex | 0.067 | 0.000 | Excellent |
| Race and ethnicity | 0.353 | 0.004 | Excellent |
| Education | 0.272 | 0.002 | Excellent |
| Income (% of FPL) | 0.350 | 0.007 | Excellent |
| Marital status | 0.135 | 0.002 | Excellent |
| Metropolitan residence | 0.260 | 0.003 | Excellent |
| ADL limitations | 0.035 | 0.002 | Excellent |
| Dual eligibility | 0.284 | 0.002 | Excellent |
| Heart failure | 0.002 | 0.000 | Excellent |
| Diabetes | 0.092 | 0.001 | Excellent |
| Hypertension | 0.069 | 0.001 | Excellent |
| Stroke | 0.013 | 0.002 | Excellent |
| Emphysema/asthma/COPD | 0.011 | 0.001 | Excellent |
| Hardening of arteries | 0.034 | 0.001 | Excellent |
| Mental disorder | 0.002 | 0.000 | Excellent |
| Osteoporosis | 0.003 | 0.001 | Excellent |
| Cancer | 0.030 | 0.001 | Excellent |
| Complete/partial paralysis | 0.015 | 0.001 | Excellent |
| Survey year | 0.137 | 0.003 | Excellent |

*Standardized mean differences (SMDs) computed using the multinomial generalization (Yang & Dalton, 2012) for multi-level categorical variables and the binary form for two-level variables. All post-IPTW SMDs are below 0.05 in the overall sample, indicating excellent balance.*

***Panel B: Beneficiaries With ADRD***

| **Covariate** | **Unweighted SMD** | **IPTW-Weighted SMD** | **Balance** |
| --- | --- | --- | --- |
| Age | 0.028 | 0.004 | Excellent |
| Sex | 0.113 | 0.032 | Excellent |
| Race and ethnicity | 0.364 | 0.052 | Good |
| Education | 0.173 | 0.128 | Imbalanced |
| Income (% of FPL) | 0.280 | 0.203 | Imbalanced |
| Marital status | 0.198 | 0.110 | Imbalanced |
| Metropolitan residence | 0.296 | 0.008 | Excellent |
| ADL limitations | 0.077 | 0.089 | Good |
| Dual eligibility | 0.225 | 0.123 | Imbalanced |
| Heart failure | 0.042 | 0.070 | Good |
| Diabetes | 0.081 | 0.003 | Excellent |
| Hypertension | 0.047 | 0.025 | Excellent |
| Stroke | 0.038 | 0.034 | Excellent |
| Emphysema/asthma/COPD | 0.108 | 0.121 | Imbalanced |
| Hardening of arteries | 0.026 | 0.079 | Good |
| Mental disorder | 0.051 | 0.036 | Excellent |
| Osteoporosis | 0.010 | 0.033 | Excellent |
| Cancer | 0.026 | 0.077 | Good |
| Complete/partial paralysis | 0.029 | 0.060 | Good |
| Survey year | 0.168 | 0.026 | Excellent |

*Because the propensity score model was estimated on the pooled analytic sample, the stabilized IPTW weights are designed to achieve balance in the full sample (Panel A). When those same pooled weights are applied within the ADRD stratum (N = 1,928), a small number of covariates retain residual imbalance (SMD 0.10-0.20) — a known feature of pooled-model IPTW when subgroups are much smaller than the full sample. All main-paper estimates use survey-weighted outcome regressions on the full sample, where balance holds; the residual within-stratum imbalance would need to have a much larger association with the outcomes than the magnitudes observed to alter the substantive conclusions, and the E-value sensitivity analysis (eTable 2) supports the robustness of the primary findings to unmeasured confounding of comparable magnitude.*

***Panel C: Beneficiaries Without ADRD***

| **Covariate** | **Unweighted SMD** | **IPTW-Weighted SMD** | **Balance** |
| --- | --- | --- | --- |
| Age | 0.046 | 0.000 | Excellent |
| Sex | 0.064 | 0.002 | Excellent |
| Race and ethnicity | 0.351 | 0.006 | Excellent |
| Education | 0.275 | 0.007 | Excellent |
| Income (% of FPL) | 0.353 | 0.010 | Excellent |
| Marital status | 0.133 | 0.004 | Excellent |
| Metropolitan residence | 0.258 | 0.003 | Excellent |
| ADL limitations | 0.029 | 0.003 | Excellent |
| Dual eligibility | 0.286 | 0.008 | Excellent |
| Heart failure | 0.007 | 0.004 | Excellent |
| Diabetes | 0.092 | 0.001 | Excellent |
| Hypertension | 0.069 | 0.002 | Excellent |
| Stroke | 0.007 | 0.005 | Excellent |
| Emphysema/asthma/COPD | 0.005 | 0.008 | Excellent |
| Hardening of arteries | 0.039 | 0.004 | Excellent |
| Mental disorder | 0.003 | 0.003 | Excellent |
| Osteoporosis | 0.002 | 0.001 | Excellent |
| Cancer | 0.033 | 0.002 | Excellent |
| Complete/partial paralysis | 0.017 | 0.003 | Excellent |
| Survey year | 0.137 | 0.004 | Excellent |

*All post-IPTW SMDs are below 0.02 in the non-ADRD stratum, indicating excellent balance.*

**eTable 2. E-Value Sensitivity Analysis for Unmeasured Confounding**

| **outcome** | **stratum** | **TM %** | **MA-TM diff (95% CI)** | **Approx RR** | **E-value (point)** | **E-value (CI)** |
| --- | --- | --- | --- | --- | --- | --- |
| Any medical practitioner visit | With ADRD | 92.7% | -1.2 pp (-4.1, +1.6) | 0.987 | 1.13 | 1.00 |
| Any medical practitioner visit | Without ADRD | 91.6% | -0.2 pp (-0.9, +0.4) | 0.997 | 1.05 | 1.00 |
| Any emergency department visit | With ADRD | 37.1% | +0.1 pp (-5.2, +5.4) | 1.003 | 1.06 | 1.00 |
| Any emergency department visit | Without ADRD | 20.8% | +0.0 pp (-0.9, +0.9) | 1.000 | 1.02 | 1.00 |
| Any outpatient hospital visit | With ADRD | 29.8% | -1.7 pp (-6.6, +3.4) | 0.944 | 1.31 | 1.00 |
| Any outpatient hospital visit | Without ADRD | 31.1% | -2.6 pp (-3.7, -1.6) | 0.917 | 1.40 | 1.29 |
| Home health visits | With ADRD | 35.8% | -3.8 pp (-9.2, +1.7) | 0.893 | 1.48 | 1.00 |
| Home health visits | Without ADRD | 7.5% | -0.9 pp (-1.5, -0.3) | 0.878 | 1.54 | 1.26 |
| Any prescription drug use | With ADRD | 98.7% | -0.2 pp (-1.5, +1.0) | 0.998 | 1.05 | 1.00 |
| Any prescription drug use | Without ADRD | 95.6% | +0.2 pp (-0.4, +0.6) | 1.002 | 1.04 | 1.00 |
| Any specialist visit | With ADRD | 61.2% | +5.2 pp (-0.1, +10.2) | 1.085 | 1.39 | 1.00 |
| Any specialist visit | Without ADRD | 68.2% | -1.1 pp (-2.1, +0.1) | 0.984 | 1.14 | 1.00 |
| Any hospital admission | With ADRD | 25.1% | -3.1 pp (-8.7, +2.7) | 0.876 | 1.54 | 1.00 |
| Any hospital admission | Without ADRD | 15.4% | -0.4 pp (-1.4, +0.4) | 0.971 | 1.21 | 1.00 |
| Dental care visits | With ADRD | 11.1% | -0.9 pp (-3.9, +2.4) | 0.920 | 1.40 | 1.00 |
| Dental care visits | Without ADRD | 8.5% | +0.4 pp (-0.3, +1.0) | 1.042 | 1.25 | 1.00 |

*E-values quantify the minimum association strength (on the risk ratio scale) that an unmeasured confounder would need to have with both MA enrollment and the outcome to fully explain away the observed adjusted association, above and beyond the measured covariates already balanced by IPTW (VanderWeele & Ding, 2017, Ann Intern Med 167:268-274). Larger E-values indicate greater robustness to unmeasured confounding. E-value (CI) is computed for the confidence limit closest to the null (RR = 1); when the CI already includes the null, E-value (CI) equals 1.00. Approximate risk ratios computed from the IPTW-adjusted TM proportion and the MA-TM difference.*

**eTable 3. Distribution of Analytic Weights**

| **Subgroup** | **N** | **Mean SW** | **SD SW** | **Mean final wt** | **SD final wt** |
| --- | --- | --- | --- | --- | --- |
| MA_ADRD | 954 | 0.937 | 0.323 | 2958 | 1931 |
| MA_nonADRD | 19,017 | 1.006 | 0.322 | 4138 | 2496 |
| TM_ADRD | 974 | 1.095 | 0.355 | 3968 | 2813 |
| TM_nonADRD | 23,050 | 0.993 | 0.287 | 4803 | 3413 |

*Analytic weights are the product of stabilized inverse probability of treatment weights (sw) and MCBS full-sample annual weights (PUFFWGT). All stabilized weights are within reasonable range (percentile 1 to 99 spanning approximately 0.6 to 2.3), indicating no extreme weights that could destabilize estimates.*

**eTable 4. Duals-Stratified Sensitivity for MA-TM Utilization Differences**

| **Outcome** | **Stratum** | **N (Non-dual)** | **MA-TM diff, Non-dual (pp, 95% CI)** | **N (Any dual)** | **MA-TM diff, Any dual (pp, 95% CI)** |
| --- | --- | --- | --- | --- | --- |
| Any medical practitioner visit | With ADRD | 1409 | -0.2 (-3.8 to +3.4) | 519 | -3.2 (-7.8 to +1.3) |
| Any medical practitioner visit | Without ADRD | 37093 | -0.2 (-0.9 to +0.5) | 4974 | -0.5 (-2.5 to +1.6) |
| Any emergency department visit | With ADRD | 1409 | +2.1 (-3.8 to +8.0) | 519 | -3.8 (-14.7 to +7.0) |
| Any emergency department visit | Without ADRD | 37093 | -0.3 (-1.2 to +0.7) | 4974 | +1.3 (-1.9 to +4.4) |
| Any outpatient hospital visit | With ADRD | 1409 | -2.4 (-8.3 to +3.5) | 519 | -1.6 (-10.5 to +7.3) |
| Any outpatient hospital visit | Without ADRD | 37093 | -2.8 (-3.9 to -1.7) | 4974 | -0.3 (-3.3 to +2.7) |
| Home health visits | With ADRD | 1409 | -4.5 (-10.5 to +1.4) | 519 | +2.8 (-8.5 to +14.1) |
| Home health visits | Without ADRD | 37093 | -0.6 (-1.1 to -0.0) | 4974 | -5.2 (-8.1 to -2.3) |
| Any prescription drug use | With ADRD | 1409 | +0.1 (-1.5 to +1.6) | 519 | -1.1 (-3.5 to +1.2) |
| Any prescription drug use | Without ADRD | 37093 | +0.1 (-0.5 to +0.7) | 4974 | +0.4 (-0.9 to +1.8) |
| Any specialist visit | With ADRD | 1409 | +4.1 (-1.8 to +10.0) | 519 | +5.4 (-5.7 to +16.5) |
| Any specialist visit | Without ADRD | 37093 | -1.7 (-2.8 to -0.5) | 4974 | +6.3 (+2.5 to +10.0) |
| Any hospital admission | With ADRD | 1409 | -0.9 (-6.7 to +5.0) | 519 | -7.6 (-19.6 to +4.3) |
| Any hospital admission | Without ADRD | 37093 | -0.5 (-1.4 to +0.5) | 4974 | -1.8 (-5.3 to +1.8) |
| Dental care visits | With ADRD | 1409 | -0.7 (-4.6 to +3.3) | 519 | -1.3 (-7.6 to +5.0) |
| Dental care visits | Without ADRD | 37093 | +0.4 (-0.3 to +1.0) | 4974 | -1.1 (-3.9 to +1.8) |

*IPTW-adjusted MA-TM differences (percentage points with 95% CIs) estimated separately among non-dual beneficiaries and any-dual (part-year or full-year) beneficiaries, within each ADRD stratum. Directional consistency across dual-eligibility strata supports the main analysis of pooling all beneficiaries with dual eligibility included as a covariate in propensity and outcome models.*

**eTable 5. Logistic Regression Sensitivity for MA-TM Utilization Differences**

***Stratified Estimates***

| **Outcome** | **With ADRD: OR (95% CI)** | **Without ADRD: OR (95% CI)** | **With ADRD: AME (pp)** | **Without ADRD: AME (pp)** |
| --- | --- | --- | --- | --- |
| Any medical practitioner visit | 0.85 (0.62-1.16) | 0.97 (0.90-1.04) | -1.2 (-3.5 to +1.1) | -0.2 (-0.8 to +0.3) |
| Any emergency department visit | 1.00 (0.85-1.19) | 1.00 (0.96-1.04) | +0.1 (-3.8 to +4.0) | +0.0 (-0.7 to +0.7) |
| Any outpatient hospital visit | 0.92 (0.77-1.11) | 0.88 (0.83-0.94) | -1.7 (-5.4 to +2.0) | -2.6 (-3.8 to -1.3) |
| Home health visits | 0.84 (0.69-1.03) | 0.87 (0.80-0.94) | -3.8 (-8.3 to +0.7) | -0.9 (-1.4 to -0.4) |
| Any prescription drug use | 0.84 (0.38-1.88) | 1.04 (0.93-1.16) | -0.2 (-1.4 to +0.9) | +0.2 (-0.3 to +0.6) |
| Any specialist visit | 1.25 (1.04-1.51) | 0.95 (0.90-1.01) | +5.2 (+1.0 to +9.5) | -1.1 (-2.3 to +0.2) |
| Any hospital admission | 0.84 (0.67-1.06) | 0.97 (0.92-1.02) | -3.1 (-7.2 to +1.0) | -0.4 (-1.1 to +0.2) |
| Dental care visits | 0.91 (0.69-1.21) | 1.05 (0.98-1.12) | -0.9 (-3.6 to +1.8) | +0.4 (-0.2 to +0.9) |

*Survey-weighted quasibinomial logistic regression stratified by ADRD status, using stabilized IPTW × MCBS sampling weights. Odds ratios (OR) with 95% CIs. Average marginal effects (AME) in percentage points computed by predicting outcome probability at MA=1 vs MA=0 for each subject and averaging the weighted difference.*

***MA × ADRD Interaction from Pooled Logistic Model***

| **Outcome** | **AME nonADRD (pp)** | **AME ADRD (pp)** | **Interaction OR (95% CI)** | **AME diff (pp)** | **P-int** |
| --- | --- | --- | --- | --- | --- |
| Any medical practitioner visit | -0.2 | -1.2 | 0.87 (0.63-1.21) | -1.0 pp | 0.415 |
| Any emergency department visit | +0.0 | +0.1 | 1.00 (0.84-1.20) | +0.1 pp | 0.961 |
| Any outpatient hospital visit | -2.6 | -1.7 | 1.04 (0.86-1.26) | +0.9 pp | 0.666 |
| Home health visits | -0.9 | -3.8 | 0.97 (0.78-1.20) | -2.9 pp | 0.779 |
| Any prescription drug use | +0.2 | -0.2 | 0.81 (0.36-1.82) | -0.4 pp | 0.613 |
| **Any specialist visit** | -1.1 | +5.2 | **1.32 (1.09-1.59)** | +6.3 pp | **0.004** |
| Any hospital admission | -0.4 | -3.1 | 0.87 (0.69-1.10) | -2.7 pp | 0.237 |
| Dental care visits | +0.4 | -0.9 | 0.87 (0.65-1.16) | -1.3 pp | 0.344 |

*Interaction odds ratio from a pooled logistic model with MA × ADRD interaction term. AME difference is the difference between stratum-specific average marginal effects. The specialist × ADRD interaction identified in the primary linear probability model analysis (Table 3) is confirmed under logistic specification (interaction OR 1.32, 95% CI 1.09-1.59, p = 0.004; AME difference +6.3 percentage points), supporting the robustness of this effect modification finding to model choice.*

**eTable 6. Variable Source and Definition Table**

| **Variable** | **Type** | **Definition** |
| --- | --- | --- |
| PUF_ID | Administrative | Beneficiary ID |
| SURVEYYR | Administrative | Survey year (2018-2022) |
| INS_D_MADV | Administrative | MA vs TM enrollment (derived) |
| ADM_MA_FLAG_YR | Administrative | MA enrollment: No / Part-year / Full-year |
| ADM_FFS_FLAG_YR | Administrative | FFS enrollment: No / Part-year / Full-year |
| ADM_H_MEDSTA | Administrative | Entitlement basis: Aged / Disabled / ESRD |
| ADM_DUAL_FLAG_YR | Administrative | Dual eligibility: Non-dual / Part-year / Full-year |
| INT_SPPROXY | Administrative | Interview respondent: Self / Proxy |
| HLT_ALZDEM | Self- or proxy-report | 'Has a doctor ever told you that you had AD or dementia?' |
| DEM_AGE | Administrative | Age category (65-74, 75+) |
| DEM_SEX | Administrative | Sex |
| DEM_RACE | Administrative | Race/ethnicity (4-level) |
| DEM_EDU | Self-report | Educational attainment |
| DEM_IPR_IND | Derived | Income relative to FPL |
| DEM_MARSTA | Self-report | Marital status |
| DEM_CBSA | Administrative | Metropolitan vs non-metropolitan |
| HLT_FUNC_LIM | Self- or proxy-report | ADL/IADL limitations count |
| HLT_OC* (10 vars) | Self- or proxy-report | 'Has a doctor ever told you...' comorbidities |
| ACW_* (8 outcomes) | Self- or proxy-report | Any use / need in past 12 months (binary) |

*MCBS Public Use File sources listed by seasonal supplement (Fall or Winter). Comorbidity variables (HLT_OC*) include HLT_OCCFAIL (heart failure), HLT_OCBETES (diabetes), HLT_OCHBP (hypertension), HLT_OCSTROKE (stroke), HLT_OCEMPHYS (emphysema/asthma/COPD), HLT_OCARTERY (hardening of arteries), HLT_OCPSYCHO (mental disorder), HLT_OCOSTEOP (osteoporosis), HLT_OCCANCER (cancer), and HLT_OCPPARAL (paralysis). Utilization outcomes (ACW_*) include ACW_PROVYR (medical practitioner visit), ACW_D_ERVIST (ER visit), ACW_D_OPVIST (outpatient hospital visit), ACW_HLTHSRVC (Home health visits), ACW_ANYRX (any prescription drug use), ACW_CARESPCL (specialist visit), ACW_HOSADMIT (hospital admission), and ACW_DVNEED (Dental care visits).*

**eFigure 1. Study Flow Diagram (CONSORT)**

*Study flow diagram showing derivation of the analytic sample from the pooled 2018-2022 MCBS Fall+Winter merged data. The final sample of 43,995 beneficiaries includes dual-eligible beneficiaries per Editor comment E1 and reflects strict enforcement of the 12-month continuous Medicare Part A + B enrollment criterion.*

**eFigure 2. Propensity Score Distributions by Medicare Plan Type**

*Density plots of estimated propensity scores (Pr(MA | X)) for Medicare Advantage (gray dashed) and Traditional Medicare (black solid) beneficiaries. Substantial overlap across the propensity score distribution supports the positivity assumption for IPTW estimation.*

**eFigure 3. Love Plot: Covariate Balance Before and After IPTW**

*Absolute standardized mean differences (SMDs) for each covariate before (gray circles) and after (black filled circles) inverse probability of treatment weighting. Vertical dashed line marks the 0.10 balance threshold. All covariates achieve balance well below 0.10 after IPTW.*
